# Novel Anti-FOLR1 Antibody–Drug Conjugate MORAb-202 in Breast Cancer and Non-Small Cell Lung Cancer Cells

**DOI:** 10.3390/antib10010006

**Published:** 2021-02-01

**Authors:** Yuki Matsunaga, Toshimitsu Yamaoka, Motoi Ohba, Sakiko Miura, Hiroko Masuda, Takafumi Sangai, Masafumi Takimoto, Seigo Nakamura, Junji Tsurutani

**Affiliations:** 1 Department of Breast Surgical Oncology, School of Medicine, Showa University, 1-5-8 Hatanodai, Shinagawa-ku, Tokyo 142-8666, Japan; yukimatsu@med.showa-u.ac.jp (Y.M.); hmasuda@med.showa-u.ac.jp (H.M.); seigonak@med.showa-u.ac.jp (S.N.); 2 Advanced Cancer Translational Research Institute, Showa University, 1-5-8 Hatanodai, Shinagawa-ku, Tokyo 142-8555, Japan; motoi-o@ims.u-tokyo.ac.jp (M.O.); tsurutaj@med.showa-u.ac.jp (J.T.); 3 Department of Pathology, School of Medicine, Showa University, Tokyo 142-8666, Japan; tazawa-shw@umin.ac.jp (S.M.); takimoto@med.showa-u.ac.jp (M.T.); 4 Department of Breast and Thyroid Surgery, School of Medicine, Kitasato University, Kanagawa 252-0375, Japan; sangai-jpn@umin.net

**Keywords:** breast cancer, non-small cell lung cancer, FOLR1, MORAb-202, eribulin

## Abstract

Antibody–drug conjugates (ADCs), which are currently being developed, may become promising cancer therapeutics. Folate receptor α (FOLR1), a glycosylphosphatidylinositol-anchored membrane protein, is an attractive target of ADCs, as it is largely absent from normal tissues but is overexpressed in malignant tumors of epithelial origin, including ovarian, lung, and breast cancer. In this study, we tested the effects of novel anti-FOLR1 antibody–eribulin conjugate MORAb-202 in breast cancer and non-small cell lung cancer (NSCLC) cell lines. FOLR1 expression, cell proliferation, bystander killing effects, and apoptosis were evaluated in seven breast cancer and nine NSCLC cell lines treated with MORAb-202. Tumor growth and FOLR1 expression were assessed in T47D and MCF7 orthotopic xenograft mouse models after a single intravenous administration of MORAb-202 (5 mg/kg). MORAb-202 was associated with inhibited cell proliferation, with specific selectivity toward FOLR1-expressing breast cancer cell lines. Eribulin, the payload of MORAb-202, was unleashed in HCC1954 cells, diffused into intercellular spaces, and then killed the non-FOLR1-expressing MCF7 cells in co-culture systems. In orthotopic xenograft mouse models, FOLR1-expressing T47D tumors and non-FOLR1-expressing MCF7 tumors were suppressed upon MORAb-202 administration. The novel anti-FOLR1 antibody–eribulin conjugate MORAb-202 has potential antitumor effects in breast cancer.

## 1. Introduction

Antibody–drug conjugates (ADCs) represent a developing class of drugs with the potential to become key anticancer therapeutics [1,2,3]. These drugs are composed of a monoclonal antibody (mAb) conjugated to a cytotoxic payload via a chemical linker and are tailored to highly specific target antigens expressed on cancer cell surfaces. Theoretically, the use of ADCs results in reduced systemic exposure to toxicity relative to chemotherapy. Moreover, ADCs are capable of delivering highly potent drugs to tumor cells while sparing normal cells, thus attenuating the clinical obstacles of traditional chemotherapy [4].

MORAb-202 is an ADC comprising the antibody farletuzumab, which targets folate receptor α (FRα, known as FOLR1), and a cathepsin-cleavable linker with eribulin as a payload. The drug-to-antibody ratio of 4:1 shows optimal biophysical properties and potent cytotoxic effects for targeting FOLR1-expressing cell lines and yielding durable responses in FOLR1-expressing xenograft models [5]. MORAb-202 was developed in a clinical phase I study for patients with FOLR1-expressing advanced solid tumors (ClinicalTrials.gov Identifier: NCT03386942).

FOLR1 is a glycosylphosphatidylinositol (GPI)-anchored membrane protein and shows a high affinity for binding and coordinating the transport of the active form of folate [6,7]. Although largely absent from normal tissues, FOLR1 has been reported to be overexpressed specifically in malignant tumors of epithelial origin, including ovarian, lung, and breast cancer [8,9]. Moreover, the expression of FOLR1 is known to be associated with the malignant potential of cancer [10]. A humanized anti-human FOLR1 mAb, farletuzumab, was evaluated in clinical trials for patients with platinum-sensitive ovarian cancer [11,12]. Although the phase III trial did not meet its primary statistical endpoint of progression-free survival, the addition of farletuzumab to standard chemotherapy for patients with relapsed platinum-sensitive ovarian cancer did not increase toxicity [13]. As a result of its strong safety profile in clinical trials and its specific ability to target FOLR1-positive tumors [14], farletuzumab is an attractive antibody for use in ADCs targeting FOLR1-positive cancer patients.

Originally isolated from the rare Japanese marine sponge *Halichondria okadai* [15], eribulin is a synthetic analog of the marine natural product halichondrin B, which interferes with microtubule dynamics. This compound displays a unique mechanism of suppressing microtubule growth without shortening the microtubule, but rather by sequestering tubulin into nonfunctional aggregates, resulting in irreversible G2-M arrest and apoptosis [16]. In addition to the anti-mitotic effect of eribulin, it has been shown to inhibit cell migration, decrease circulating vascular endothelial growth factor, and promote mesenchymal–epithelial transition in tumor phenotypes [17,18,19]. Moreover, the adverse effect of peripheral neuropathy has been clinically demonstrated to be lower under eribulin treatment than under treatment with other microtubule-targeting agents such as paclitaxel and ixabepilone [20,21]. In the EMBRACE (eribulin mesylate versus treatment of physician’s choice) trial, a phase III study, eribulin exhibited a statistically significant survival benefit in pretreated patients with locally advanced or metastatic breast cancer [22]. Eribulin is used for the treatment of patients with advanced or metastatic breast cancer who are refractory to other forms of treatment [23] and is an interesting candidate as a payload for an ADC.

The linker plays an important role in an ADC, linking the cytotoxic drug to the mAb. MORAb-202 is characterized by a cleavable linker, which is lysosomal protease-sensitive. A lysosomal protease, such as cathepsin, is more highly expressed in tumor cells than in normal cells [24]. Therefore, after selectively binding to FOLR1 and being transported into cancer cells through receptor-mediated endocytosis, the linker unleashes its cytotoxic payload, eribulin, as a result of lysosomal protease cleavage. The unleashed eribulin eradicates the FOLR1-expressing cell and then penetrates surrounding tissues, killing other cancer cells in what is known as the bystander killing effect. Therefore, MORAb-202 might be beneficial in the treatment of tumors expressing heterogeneous targets [25].

In this study, we tested the effect of MORAb-202, a novel anti-FOLR1 antibody–eribulin conjugate, in breast cancer and non-small cell lung-cancer (NSCLC) cell lines. MORAb-202 exhibited an inhibition of cell proliferation with specific selectivity toward FOLR1-expressing breast cancer cell lines but not FOLR1-expressing NSCLC lines. Eribulin was unleashed in the cells, resulting in G2-M cell cycle arrest, and then went on to kill the non-FOLR1-expressing neighboring cells in co-culture systems. Interestingly, in orthotopic xenograft mouse models, not only was the FOLR1-expressing T47D tumor suppressed upon MORAb-202 administration but the non-FOLR1-expressing MCF7 tumor was also completely suppressed; we attribute the latter result to the induction of FOLR1 in vivo. These results warrant further investigations into the effect of MORAb-202 on FOLR1-expressing and non-expressing cancer cells.

## 2. Materials and Methods

### 2.1. Cell Lines and Reagents

ER-positive/HER2-negative breast cancer cell lines MCF7 and T47D, ER-positive/HER2-positive breast cancer cell lines MDA-MB-361 and SKBR3, ER-negative/HER2-positive breast cancer cell line HCC1954, and TNBC cell lines MDA-MB-231 and BT549 were purchased from American Type Culture Collection (Manassas, VA, USA). Lung cancer cell lines, HCC827, NCI-H1650, HCC4006, NCI-H1975, A549, NCI-H441 and BEAS-2B were purchased from American Type Culture Collection (Manassas, VA, USA). The PC-14 cell line was obtained from IBL (Gunma, Japan), the PC-9 cell line was donated by K. Hayata (Tokyo Medical College, Tokyo, Japan) during the 1980s, and the ABC-1 line was obtained from JCRB Cell Bank. Cell lines used in this study were authenticated by short tandem-repeat analysis at the Japanese Collection of Research Bioresources cell bank in 2019 and tested for mycoplasma using a MycoAlert Mycoplasma Detection Kit (Lonza; Rockland, ME, USA) in 2019. MDA-MB-361 and MDA-MB-231 were maintained in Dulbecco’s Modified Eagle Medium (DMEM) with 10% fetal bovine serum (FBS) and penicillin (100 U/mL), and streptomycin (100 μg/mL). MCF7, T47D, HCC1954, PC-9, HCC827, NCI-H1650, NCI-H1975, PC-14, A549, NCI-H441, ABC-1, and BEAS-2B were cultured in Roswell Park Memorial Institute (RPMI)-1640 medium supplemented with 10% FBS and penicillin (100 U/mL), and streptomycin (100 μg/mL). HCC4006 was cultured in RPMI-1640 medium supplemented with 5% FBS and penicillin (100 U/mL), and streptomycin (100 μg/mL). SK-BR-3 was maintained in McCoy’s 5A medium with 10% FBS and penicillin (100 U/mL), and streptomycin (100 μg/mL). Cells were cultured at 37 °C in a humidified 5% CO_2_ atmosphere. Cells were passaged for <4 months prior to renewal from the frozen stock. MORAb-202 was kindly provided by Eisai Inc. (Cambridge, MA, USA). Eribulin was purchased from Showa University Hospital (Tokyo, Japan). Other chemicals and cell culture media were purchased from FUJIFILM Wako Pure Chemical Corporation (Osaka, Japan).

### 2.2. Real-Time RT-PCR

Total RNA was isolated from cells using an RNeasy Mini Kit (Qiagen; Tokyo, Japan) and cDNA was synthesized from each isolated RNA sample using random 6-mers and an RT-PCR Kit (Takara Bio; Kyoto, Japan). cDNA samples were amplified and analyzed using the PowerUP SYBR Green PCR master mix (Applied Biosystems; Waltham, MA, USA) and a fluorescence-based RT-PCR-detection system (GeneAmp 5700; Applied Biosystems; Foster City, CA, USA). The *FOLR1* gene was amplified using the following PCR primers: forward, 5′-CTTAGCCTGGCCCTAATGCT-3′ and reverse, 5′-GCTGAACAGGGCAGGGATTT-3′. *GAPDH* gene was amplified as an internal control using the following primers: forward, 5′-TGCACCACCAACTGCTTAG-3′ and reverse, 5′-GGCATGGACTGTGGTCATGAG-3′.

### 2.3. Cell Proliferation Assay

Cells were plated in 96-well plates at densities of 2000 to 4000 cells per well, depending on the growth rate of each cell line. Cells had adhered overnight (16–24 h) before being cultured with indicated concentrations of MORAb-202 for 4 days at 37 °C in a humidified atmosphere containing 5% CO_2_. Then, cell number and viability were measured by CellTiter 96 Non-Radioactive Cell Proliferation Assay (Promega; Madison, WI, USA). The optical density was measured at 570 nm (OD570) with a Power scan HT microplate reader (BioTek; Winooski, VA, USA). We prepared six replicates, and the experiments were repeated at least three times. The percentage of the values obtained from the control cells was displayed graphically, and the IC_50_ values were determined using GraphPad Prism version 8 (GraphPad, Inc.; San Diego, CA, USA).

### 2.4. Western Blot Analysis

Cells were washed with phosphate-buffered saline (PBS) and lysed with RIPA Buffer (20 mM Tris [pH 7.5], 1 mM ethylenediaminetetraacetic acid (EDTA), 150 mM NaCl, 1% Triton, 0.1% SDS, 0.5% deoxycholic acid). The protein was quantified using a BCA Protein Assay Kit (ThermoFisher; Waltham, MA, USA). Equal amounts of protein samples were separated by 10% SDS-PAGE electrophoresis and transferred onto polyvinylidene fluoride (PVDF) membranes (Merck Millipore; Burlington, MA, USA). Membranes were blocked with 5% skim milk for 1 h. Immunoblotting was performed by incubating the membranes overnight with FOLR1 antibody (60307-1-1; Proteintech; Rosemont, IL, USA), and β-actin antibody (4970; Cell Signaling Technologies; Danvers, MA, USA) as primary antibodies (diluted with Tris Buffered Saline with Tween 20 (TBST) according to manufacturer’s instructions, 1:1000 to 1:2000). After washing with TBS-0.05% Tween, membranes were incubated with anti-mouse immunoglobulin (Ig)G, HRP-linked antibody (7076; Cell Signaling Technologies Danvers, MA, USA) or anti-rabbit IgG, and horseradish peroxidase (HRP)-linked antibody (7074; Cell signaling Technologies) as secondary antibodies (diluted with TBST according to manufacturer’s instructions, 1:1000 to 1:2000). After washing with TBS-0.05% Tween, membranes were soaked with LuminataTM Crescendo Western HRP Substrate (Merck Millipore, Burlington, MA, USA) and visualized. β-actin was used as a loading control, and human FOLR1 transfected/overexpressed HEK293 cellular lysate was used as a positive control (Sino Biological Inc.; Wayne, PA, USA).

### 2.5. RNA Interference

Non-targeting (NT) siRNA and SMARTpool siRNAs targeting *FOLR1* (M010403) were purchased from Dharmacon (Lafayette, CO, USA). Cells were seeded into 6-well plates in RPMI 1640 medium supplemented with 10% FBS without antibiotics. The following day, the cells were transfected with 100 pmol/well siRNA using Lipofectamine 2000 Transfection Reagent (Life Technologies; Carlsbad, CA, USA) according to the manufacturer’s instructions and were analyzed 72 h post-transfection.

### 2.6. Transwell Co-Culture Assay

The bystander activity was assessed by a co-culture of MCF-7 and HCC1954 cells in dual-chamber transwell dishes. A total of 1.25 × 10^5^ MCF-7 cells, which are not sensitive to MORAb-202, were plated in the bottom chamber of the 6-well cell culture plates, while 1.0 × 10^5^ H1954 cells were plated in the upper transwell permeable inserts (Millipore, Burlington, MA, USA), with a membrane pore size of 1.0 μm. In this model, any potential bystander factors can diffuse through the permeable membrane, but the MCF-7 cells cannot. After treatment with MORAb-202 (10 nM) or growth medium and 96 h of incubation, the cells in the bottom wells were stained with 0.005% crystal violet and counted using a TC-20 automated cell counter (Bio-Rad; Hercules, CA, USA). The data are presented as mean ± SEM of the data obtained from six replicate wells, and the results with *p* < 0.05 were considered to be statistically significant.

### 2.7. Flow Cytometric Analysis

The 70%-confluent T47D cells were treated with eribulin (0.7 nM, the 2.5× IC_50_ value) or MORAb-202 (7 nM, the 2.5× IC_50_ value) for 0, 2, 6, 12, 24, and 48 h, fixed with 100% ethanol at −20 °C overnight, and then stained with propidium iodide. Flow cytometric analysis was performed using a FACSCalibur (BD Biosciences; Franklin Lakes, NJ, USA). The cell cycle distributions were calculated using CellQuest Pro software (BD Biosciences). The data are presented as mean ± SEM of three independent experiments, and statistical significance was set at *p* < 0.05 in comparison with the data from 0 h.

### 2.8. Orthotopic Xenograft Mouse Studies

All animal experiments were performed according to protocols approved by the Institutional Animal Care Committee at Showa University (code; 59008, date 1 April 2019). Seventeen β-estradiol pellets (0.5 mg, 60-day release, Innovative Research of America) were implanted subcutaneously into female NOD/SCID or BALB/c-nu/nu nude mice. The next day, 5 × 10^6^ T47D or MCF7 cells, in 100 μL of 50% Matrigel (Fisher Scientific, Waltham, MA, USA) diluted with sterile PBS, were subcutaneously implanted into the fourth mammary fat pad bilaterally of 6-week-old female NOD/SCID (T47D cells) or BALB/c-nu/nu nude (MCF7 cells) mice, respectively. After the tumor volume reached 50–100 mm^3^, the mice were divided into two groups (*n* = 6 mice/group), namely, MORAb-202 and control. Mice in the MORAb-202 group were injected with 5 mg/kg of MORAb-202, and the mice in the control group were injected 100 μL PBS on day 1, once through the tail vein. The average volume of the subcutaneous bilateral tumors was assessed by caliper measurement twice weekly for 63 days in T47D tumors and 32 days in MCF7 tumors. The tumor volume was calculated using the following formula: length^2^ × width × 0.5. All values represent the mean ± SD of 12 tumors per group. For observing the xenografted tumor tissues of T47D and MCF7, using hematoxylin and eosin (H&E) and immunohistochemistry (IHC), for FOLR1, the T47D and MCF7 cells were implanted again. After the tumor volume reached 50–100 mm^3^, the mice were divided into two groups (*n* = 2 mice/group), namely, MORAb-202 and control. Seven days after PBS (as control) or MORAb-202 (5 mg/kg) administration, orthotopic T47D and MCF7 xenograft tumors were obtained. Sections from 4% paraformaldehyde-fixed paraffin-embedded blocks of xenograft samples were cut to a thickness of 5 μm. Slides were deparaffinized. Antigen retrieval was performed by autoclaving at 121 °C for 10 min, endogenous peroxidase activity was quenched with 1% hydrogen peroxidase/methanol; then, the sections were blocked with 5% skim milk/TBST and incubated with rabbit polyclonal antibody against folate receptor 1 (1:50 dilution, 60307-1-1; Proteintech, Rosemont, IL, USA) overnight at 4 °C. Staining was amplified by the addition of a biotinylated anti-rabbit antibody and an avidin-biotinylated HRP conjugate for 10 min (LSAB2 kit; DAKO, Santa Clara, CA, USA) and developed using 3,3′-Diaminobenzidine (DAB). Sections were counterstained with hematoxylin, dehydrated in ethanol, cleaned with xylene, and mounted. H&E staining was performed for histological diagnosis by pathologists.

### 2.9. TP53 and PIK3CA Sequence Analysis

The genomic DNA was isolated from the paraffin-embedded tumor tissues of T47D and MCF7 mice using the DNeasy Blood and Tissue kit (Qiagen, Germantown, MD, USA). Then, exon 6 of the TP53 gene and exons 9 and 20 of the PIK3CA gene were amplified from genomic DNA via PCR. The PCR products were purified and sequenced by FASMAC (Atsugi, Japan). The following primers were used for PCR and sequence analysis: exon 6 of the TP53 gene forward and reverse primers were respectively 5′-GGCCTCTGATTCCTCAGTG-3′, 5′-GCCACTGACAACCACCCTTA-3′; exon 9 of the PIK3CA gene forward and reverse primers were respectively 5′-CCAGAGGGGAAAAATATGACA-3′, 5′-CATTTTAGCACTTACCTGTGAC-3′; exon 20 of the PIK3CA gene forward and reverse primers were respectively 5′-CATTTGCTCCAAACTGACCA-3′, 5′-TGAGCTTTCATTTTCTCAGTTATCTTTTC-3′.

### 2.10. Statistical Analysis

Data are presented as means ± SEM and analyzed using GraphPad Prism version 8.0 software (GraphPad, Inc.; San Diego, CA, USA). Statistical significance was evaluated by two-tailed Student t-test and, unless otherwise noted, results with *p* < 0.05 were considered statistically significant.

## 3. Results

### 3.1. FOLR1 Expression Levels in Breast Cancer and NSCLC Cell Lines

**Figure 1:**
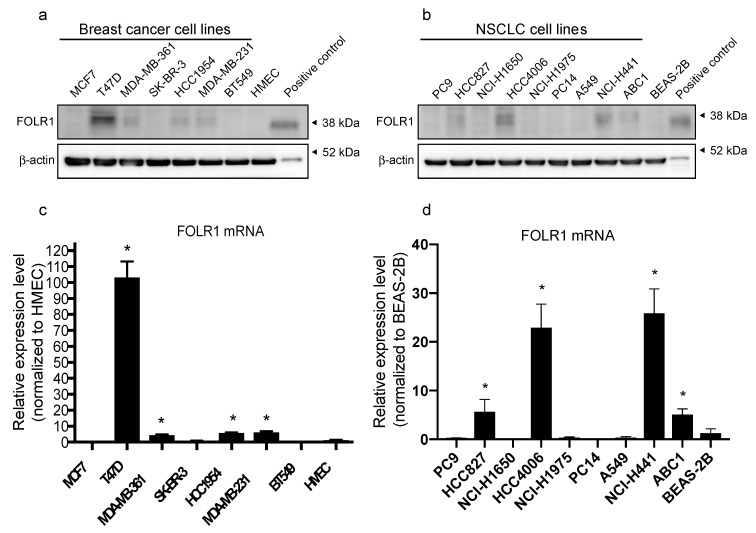
Folate receptor α (FOLR1) expression in breast cancer and non-small cell lung cancer (NSCLC) cell lines. (**a**,**b**) FOLR1 expression was determined by Western blot analysis in the breast cancer cell lines MCF7, T47D, MDA-MB-361, SK-BR-3, HCC1954, MDA-MB231, and BT549 and in the NSCLC cell lines PC-9, HCC827, NCI-H1650, HCC4006, NCI-H1975, PC-14, A549, NCI-H441, and ABC-1. Results were compared with those from normal human mammary epithelial cells (HMECs) and normal bronchial epithelial cells (BEAS-2B), respectively. Blots are representative of three independent experiments. (**c**,**d**) The cellular mRNA transcripts were quantified by real-time RT-PCR. Data are presented relative to HMEC values in breast cancer cell lines and BEAS-2B values in NSCLC cell lines, as means ± SEM (*n* = 8); * *p* < 0.01.

The protein expression levels of FOLR1 varied in both breast cancer and NSCLC cell lines. The breast cancer cell lines T47D, MDA-MB-361, HCC1954, and MDA-MB-231, and the NSCLC cell lines HCC827, HCC4006, NCI-H441, and ABC-1 showed particularly high FOLR1 expression levels (Figure 1a,b, Supplementary Figure S1). FOLR1 protein expression has been shown as multiple or broad bands because of its microheterogeneity in N-glycosylation and the presence of incompletely modified intracellular protein in the cell lysates [26,27,28]. The mRNA expression of *FOLR1* was examined by real-time RT-PCR in normal human mammary epithelial cells (HMECs) and in bronchial epithelial cells (BEAS-2B), which were used as controls for breast cancer and NSCLC cells, respectively. We found significantly elevated *FOLR1* mRNA expression in T47D, MDA-MB-361, HCC1954, and MDA-MB-231 cells (Figure 1c). The NSCLC cell lines, HCC827, HCC4006, NCI-H441, and ABC-1 exhibited significantly higher *FOLR1* expression than BEAS-2B cells (Figure 1d). Generally, FOLR1 protein and mRNA expression levels were consistent with each other.

### 3.2. The Effect of MORAb-202 on Proliferation in Breast Cancer and NSCLC Cell Lines

**Figure 2:**
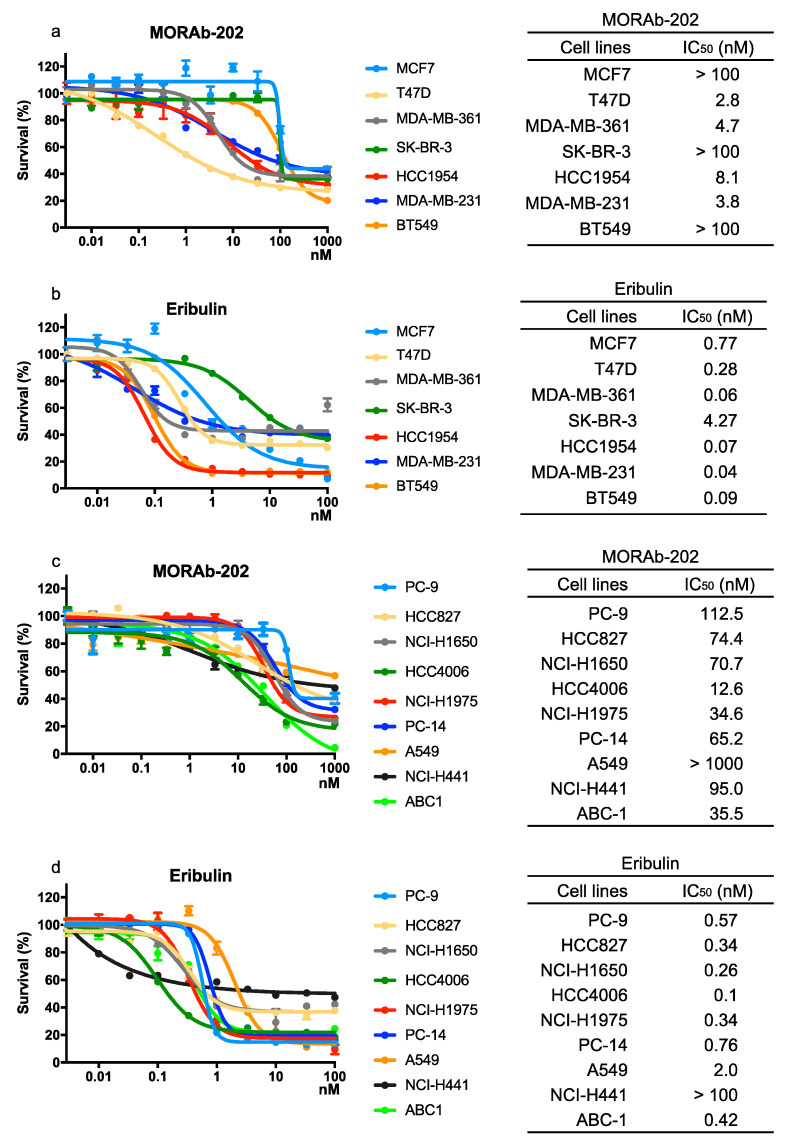
The effect of MORAb-202 and eribulin on proliferation in breast cancer and NSCLC cell lines. Cells were treated with MORAb-202 (**a**,**c**) or eribulin (**b**,**d**) at the indicated concentrations for 96 h. Cell proliferation assays were performed, OD 570 values were measured after 96 h of incubation, and the cell proliferation curves of MORAb-202 and eribulin treatments in the indicated breast and NSCLC cell lines are shown (left panels). Data represent the means ± SEMs for six wells. IC_50_ values were determined using GraphPad Prism version 8 and are expressed in the corresponding right panels.

The effect of MORAb-202 on proliferation in breast cancer and NSCLC cell lines was compared with that of eribulin, which is the payload of MORAb-202. In breast cancer cells, MORAb-202 significantly (*p* = 0.001) distinguished between sensitive and non-sensitive cell lines (Figure 2a). The sensitive cell lines, which expressed FOLR1 at high levels, were T47D, MDA-MB-361, HCC1954, and MDA-MB-231 with IC_50_ values under 10 nM. By contrast, MCF7, SK-BR-3, and BT549 cells, in which FOLR1 expression is low, were significantly resistant to MORAb-202 (*p* = 0.001). Eribulin was associated with suppressed proliferation in all breast cancer cell lines, including MCF7, SK-BR-3, and BT549 (Figure 2b). In comparison, NSCLC cell lines exhibited relative resistance to MORAb-202, with IC_50_ values over 10 nM. The high-FOLR1-expressing cell lines HCC827, HCC4006, NCI-H441, and ABC-1 exhibited moderate sensitivity to MORAb-202 (Figure 2c). Eribulin suppressed proliferation in all NSCLC cell lines regardless of FOLR1 expression (Figure 2d). MORAb-202 preferentially inhibited FOLR1-expressing breast cancer cell lines. MORAb-202 exhibited modest IC_50_ values compared to eribulin (Supplementary Figure S2) except in T47D cells, which have the highest expression of FOLR1.

### 3.3. FOLR1 Attenuation Decreases the Effect of MORAb-202 on Cell Proliferation

**Figure 3:**
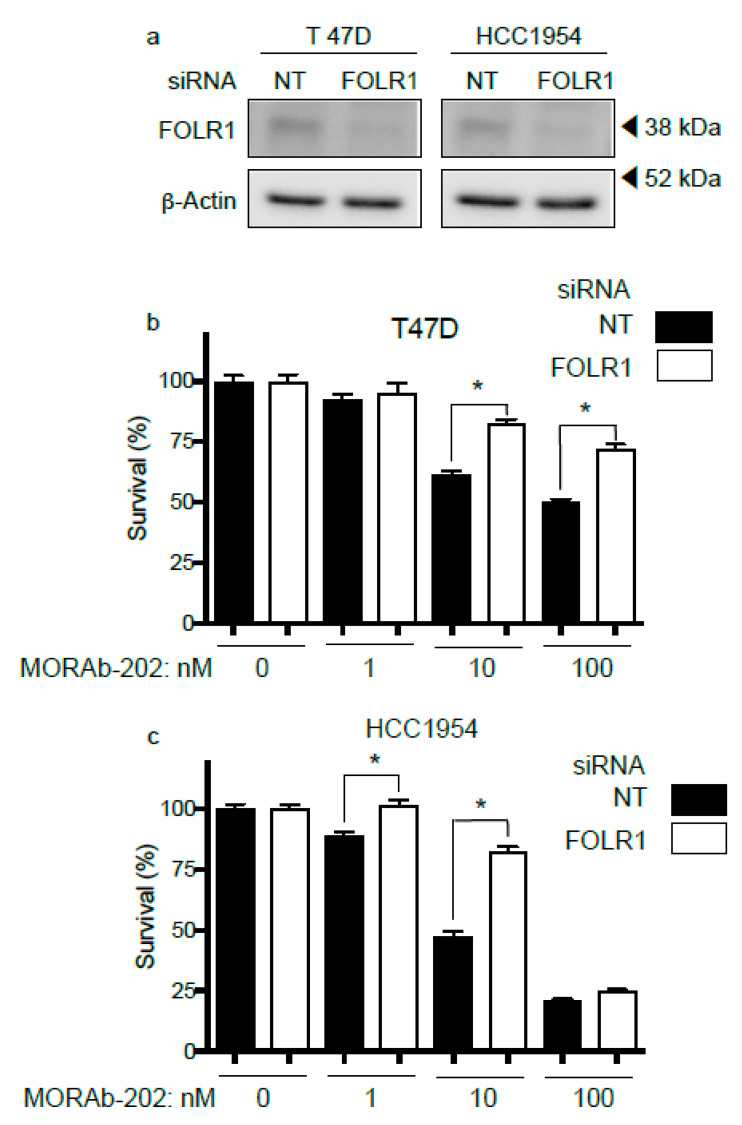
FOLR1 attenuation decreases the effect of MORAb-202 on cell proliferation. (**a**) *FOLR1* knockdown was determined by Western blot analysis. Lysates were subjected to immunoblot analysis using the indicated primary antibody. (**b**,**c**) T47D and HCC1954 cells, which are sensitive to MORAb-202, were transfected with NT siRNA or siRNA directed against *FOLR1*. Transfected cells were reseeded in the presence or absence of the indicated concentration of MORAb-202. Data represent the means ± SEMs of the data obtained from for six replicated wells; * *p* < 0.01.

MORAb-202 is an ADC that targets FOLR1. Therefore, the attenuation of FOLR1 should lead to a decrease in the cell proliferation inhibitory effect. T47D and HCC1954 cells express FOLR1 at high levels and are sensitive to MORAb-202 treatment. These cell lines were transfected with siRNA directed against FOLR1; Western blot analysis confirmed that the expression of FOLR1 was attenuated (Figure 3a). The sensitivity to MORAb-202 was reduced in both T47D and HCC1954 cells in which FOLR1 expression was attenuated (Figure 3b,c), indicating that the effect of MORAb-202 is regulated by FOLR1 expression.

### 3.4. MORAb-202 Induced G2-M Phase Cell Cycle Arrest and Accumulation of Cells in the Sub-G1 Phase

**Figure 4:**
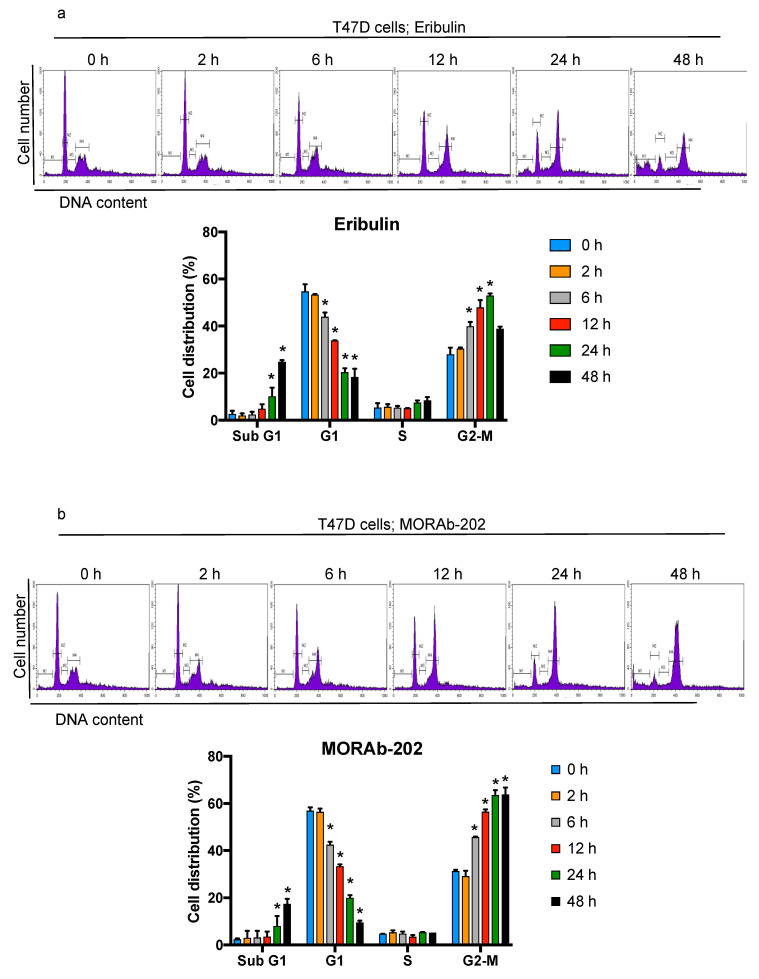
MORAb-202 increases the proportion of cells in the sub-G1 and G2-M phases. (**a**,**b**) T47D cells were cultured with eribulin (0.7 nM, which is 2.5-fold the IC_50_ value of T47D cells) or MORAb-202 (7 nM, which is 2.5-fold the IC_50_ value of T47D cells) for 0, 2, 6, 12, 24, and 48 h, followed by staining with propidium iodide and analysis of cell cycle distributions via flow cytometry. Histograms show the number of cells (vertical axis) versus the DNA content (horizontal axis) in the upper panels. In the lower panel, the percentages of cell cycle phases were calculated from the histogram. The sub-G1 phase, which indicates the apoptotic cell fraction, is Label M1; the G1, S, and G2-M phases are Labels M2, M3, and M4, respectively. Data are representative of three independent experiments; * *p* < 0.01 in comparison with data from 0 h.

T47D cells, which showed the greatest sensitivity to MORAb-202, were used to determine the effect of this drug on the cell cycle. Treatment with eribulin and MORAb-202 significantly enhanced the proportion of cells in the G2-M phase from 6 to 24 h. In addition, the accumulation of cells in the sub-G1 phase increased from 24 to 48 h upon eribulin and MORAb-202 treatment, indicating that the induction of apoptosis had occurred. The alteration of the cell cycle distribution with MORAb-202 treatment was similar to that with eribulin treatment (Figure 4a,b). These findings indicate that the linker in MORAb-202 is enzymatically cleaved in the lysosome, and eribulin is released from farletuzumab in T47D cells; then, the free eribulin induces G2-M phase arrest and the accumulation of cells in the sub-G1 phase of the cell cycle.

### 3.5. MORAb-202 Inhibited Insensitive MCF7 Cell Proliferation in Co-Culture with HCC1954 Cells

**Figure 5:**
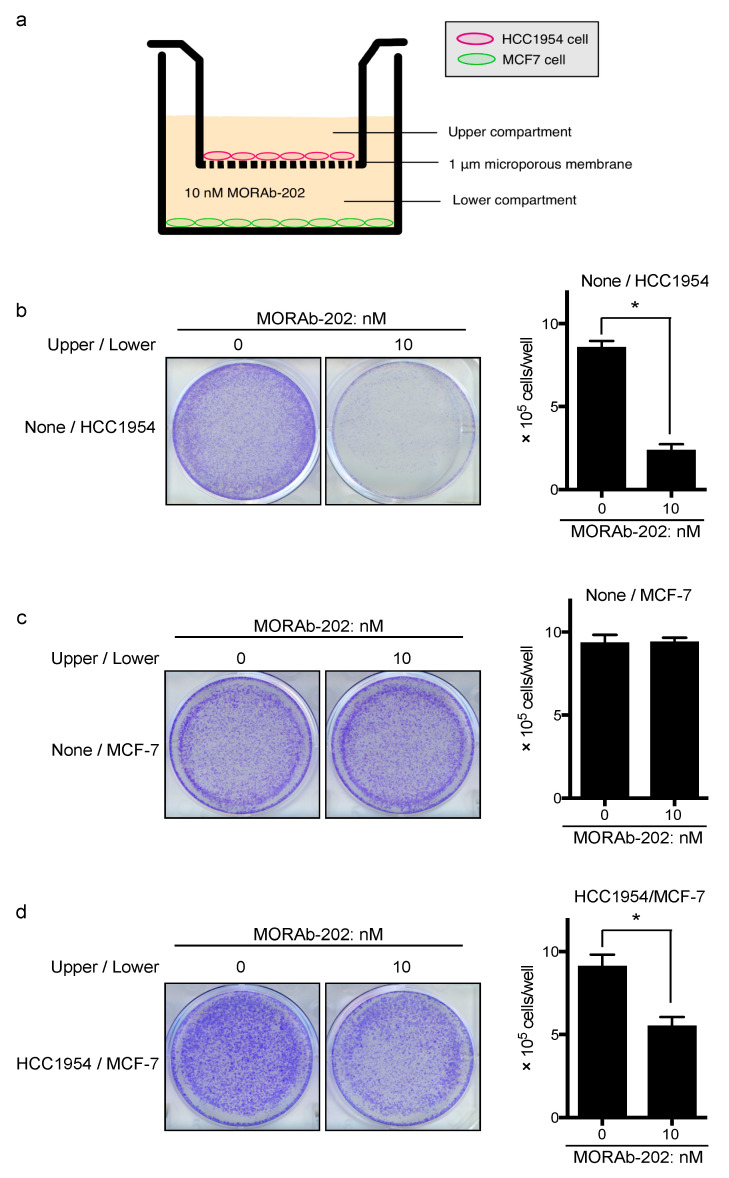
MORAb-202 inhibited the proliferation of MCF7 cells in co-culture with HCC1954 cells. (**a**) Schema of transwell co-culture system. (**b**) HCC1954 cells, which express FOLR1, were treated with or without 10 nM MORAb-202. Cells in the bottom well were stained with 0.005% crystal violet and counted. (**c**) MCF7 cells, which do not express FOLR1, were treated with or without 10 nM MORAb-202. Cells in the bottom well were stained with 0.005% crystal violet and counted. (**d**) MCF7 cells were plated in the bottom chamber of the 6-well plate, while HCC1954 cells were plated in the upper transwell permeable inserts with a membrane pore size of 1 μm, which fit into the 6-well culture plate. In this model, any potential bystander factors can diffuse through the permeable membrane. After 96 h of incubation with or without 10 nM MORAb-202, the cells in the bottom well (MCF7) were stained with 0.005% crystal violet and counted. Data represent the means ± SEMs, obtained from six replicate wells; * *p* < 0.01.

Next, we tested the bystander effect of MORAb-202 by using FOLR1-positive HCC1954 and FOLR1-negative MCF7 cells in transwell co-culture systems (Figure 5a). MCF7 cells and HCC1954 cells were seeded into the lower and upper compartments, respectively. After overnight incubation, the medium was supplemented with MORAb-202 (10 nM) in both chambers, which effectively inhibited the proliferation of HCC1954 cells but not MCF7 cells (Figure 5b,c). After 96 h, MCF7 cells in the lower compartment were stained and counted (Figure 5d). As the remaining MCF7 cells were significantly decreased in this transwell co-culture system, we suggest that free eribulin diffused from dying HCC1954 cells in the upper compartment and passed through the 1.0 μm pore size membrane to the lower compartment and killed MCF7 cells, which are sensitive to eribulin, but not to MORAb-202 (Figure 2a,b).

### 3.6. MORAb-202 Suppressed Orthotopic Xenograft Tumor Growth of T47D Cells, Which Are Sensitive to MORAb-202 In Vitro

**Figure 6:**
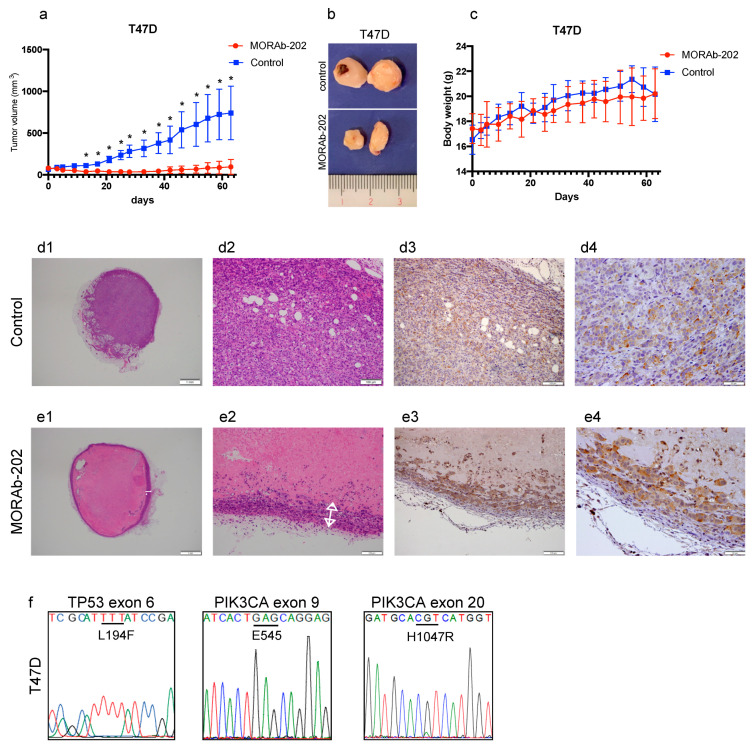
MORAb-202 suppressed orthotopic xenograft tumor growth of T47D cells. (**a**) T47D cells were orthotopically injected into the bilateral fourth mammary fat pad of NOD/SCID mice. Six mice were randomized into groups and intravenously treated with or without 5 mg/kg of MORAb-202. Tumors were measured twice weekly for 63 days. Data represent mean tumor volumes ± SEMs; * *p* < 0.01. (**b**) The representative T47D tumors after 63 days (the upper: the control, the lower: MORAb-202-treated tumor). (**c**) Body weight changes in non-obese diabetic/severe combined immune deficiency (NOD/SCID) mice over 63 days of treatment with PBS (control) and with MORAb-202 (5 mg/kg). (**d1**,**d2**,**e1**,**e2**) Seven days after PBS or MORAb-202 administration, the T47D tumor was stained with hematoxylin and eosin. Images were obtained at magnifications of 20× and 100×. The arrow indicates the peripheral tumor layer in **e1** and **e2**. (**d3**,**d4**,**e3**,**e4**) Seven days after PBS or MORAb-202 administration, FOLR1 was detected in paraffin-embedded T47D-derived tumor tissue by immunostaining at a magnification of 100× and 400×. (**f**) The sequence read of T47D tumor DNA in exon 6 TP53 and exons 9 and 20 PIK3CA.

Next, we determined the effect of MORAb-202 in an orthotopic xenograft model. After T47D cells were transplanted into the bilateral fourth mammary fat pad of NOD/SCID mice and the orthotopic T47D xenograft tumor volumes reached approximately 50–100 mm^3^, PBS (as control) or MORAb-202 was injected into the tail veins of mice on Day 1. Tumor volume and body weight were measured twice weekly. In the MORAb-202 treatment group, the orthotopic xenograft tumor growth was significantly suppressed. Interestingly, tumor growth was not observed until Day 63 (Figure 6a,b). The body weight did not differ between the control and MORAb-202 treatment groups (Figure 6c).

Seven days after PBS or MORAb-202 administration, the orthotopic xenograft tumors were removed and stained for FOLR1 by hematoxylin and eosin (H&E) and immunohistochemistry (IHC). In Figure 6d1,d2, as a control tumor of T47D cells, the solid tumor was obtained and was replete with T47D cells. FOLR1 was highly detected in T47D tumor tissue (Figure 6d3,d4). On the contrary, in MORAb-202-administered tumors, necrotized tissue was observed throughout the majority of the core area of orthotopic xenograft tumor, as shown in Figure 6e1. The T47D tumor cells, which exhibited FOLR1 expression, occurred only in the peripheral area (Figure 6e2–e4). These results demonstrate that the majority of the MORAb-202-sensitive T47D tumor was necrotized, with T47D cells remaining only in the peripheral area 7 days after MORAb-202 administration (white arrow in Figure 6e1). Elementary genetic analysis was performed by a direct sequence in TP53 exon 6 and PIK3CA in exons 9 and 20 (Figure 6f). As previously reported, in this orthotopic xenograft tumor of T47D, L194F in TP53 exon 6 and H1047R in PIK3CA exon 20 were determined; however, exon 9 PIK3CA was wild type [29,30].

### 3.7. MORAb-202 Suppressed Orthotopic Xenograft Tumor Growth of MCF7 Cells, Which Are Resistant to MORAb-202 In Vitro

**Figure 7:**
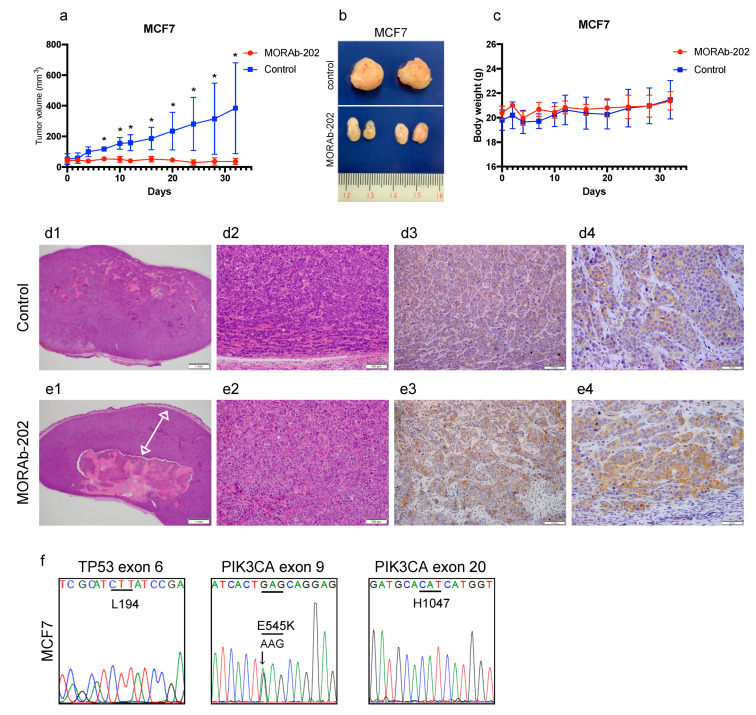
MORAb-202 suppressed orthotopic xenograft tumor growth of MCF7 cells. (**a**) MCF7 cells were orthotopically injected into the bilateral fourth mammary fat pad of BALB/c-nu/nu nude mice. Six mice per group were randomized into groups and intravenously treated with or without 5 mg/kg of MORAb-202. Tumors were measured twice weekly for 32 days. Data in the figure represent mean tumor volumes ± SEMs; * *p* < 0.01. (**b**) The representative MCF7 tumors after 32 days (the upper: the control, the lower: MORAb-202-treated tumor). (**c**) Body weight changes in BALB/c-nu/nu nude mice over 32 days of treatment with PBS as control and with MORAb-202 5 mg/kg. (**d1**,**d2**,**e1**,**e2**) Seven days after PBS or MORAb-202 administration, the MCF7 tumor was stained with hematoxylin and eosin. The images were obtained at magnifications of 20× and 100×. The arrow indicates the peripheral tumor layer in **e1**. (**d3**,**d4**,**e3**,**e4**) Seven days after PBS or MORAb-202 administration, FOLR1 was detected in paraffin-embedded MCF7-derived tumor tissue by immunostaining at a magnification 100× and 400×. (**f**) The sequence read of MCF7 tumor DNA in exon 6 TP53 and exons 9 and 20 PIK3CA.

MCF7 cells exhibited resistance to MORAb-202 in vitro (Figure 2b). Surprisingly, the in vivo orthotopic xenograft tumor growth was completely suppressed by MORAb-202 administration (Figure 7a,b). Moreover, mouse body weight did not decrease after MORAb-202 administration (Figure 7c).

Seven days after PBS or MORAb-202 administration, the orthotopic xenograft tumors were removed, followed by H&E and IHC staining of FOLR1. As described for the T47D tumor, the MCF7 tumor, which was administered PBS, was solid and replete with MCF7 cells (Figure 7d1,d2). Interestingly, diffused FOLR1 expression was detected in MCF7 orthotopic xenograft tumor tissue (Figure 7d3,d4), although FOLR1 was scarcely detected in MCF7 cells in vitro by Western blotting analysis and RT-PCR. Upon MORAb-202 administration, the peripheral MCF7 tumor volume (indicated by the white arrow in Figure 7e1) was larger than that of the T47D tumor, suggesting that MCF7 cells are relatively less sensitive to MORAb-202 than T47D cells in vivo (Figure 7e1,e2). Interestingly, FOLR1 expression was detected diffusely throughout the MCF7 orthotopic xenograft tumor tissue (Figure 7e3,e4), although FOLR1 is scarcely detected by Western blot analysis or RT-PCR for in vitro MCF7 cells. We speculated that the induction of FOLR1 in vivo was one of the reasons that the growth of MCF7 orthotopic xenograft tumors was inhibited by MORAb-202 administration. Nevertheless, the mechanisms of FOLR1 induction in MCF7 orthotopic xenograft tumors remain unclear. Similar to the T47D tumors, elementary genetic analysis was performed in TP53 and PIK3CA genes. In the MCF7 tumor, while PIK3CA exon 9 showed the E545K mutation, there were no mutations in TP53 exon 6 and PIK3CA exon 20—that is, they were wild types (Figure 7f). This indicated that the cross-contamination of T47D and MCF7 cells was excluded from the difference in the TP53 and PIK3CA mutations.

## 4. Discussion

In this study, the novel ADC MORAb-202, which comprises an anti-FOLR1 antibody and farletuzumab-linked eribulin, demonstrated a significant inhibition of proliferation in the FOLR1-expressing breast cancer cell lines. Theoretically, MORAb-202 specifically binds with and internalizes FOLR1 into intracellular spaces. Therefore, we hypothesized that MORAb-202 specifically inhibits cell proliferation and tumor growth in FOLR1-positive cells, and its effect on cell cycle distribution is similar to that of eribulin, which is a payload of MORAb-202. T47D cells, which are the breast cancer cells with the highest expression of FOLR1, exhibited a significant inhibition of cell proliferation and tumor growth, in vitro and in vivo, respectively. Moreover, G2-M cell cycle arrest and subsequent apoptosis induction were observed, as noted with eribulin [16]. Furthermore, MORAb-202 inhibited the proliferation of FOLR1-negative MCF7 cells, when co-cultured with FOLR1-positive HCC1954 cells. Therefore, the expression of FOLR1 is required to drive the cytotoxic effect of MORAb-202, although MORAb-202 also has heterogeneous targets owing to its eribulin payload.

The ADCs that are approved for clinical use include ado-trastuzumab emtansine (T-DM1) and trastuzumab deruxtecan (T-DXd, formerly DS-8201a). These ADCs consist of anti-human epidermal growth factor receptor 2 (HER2) antibodies and either the trastuzumab-linked tubulin-targeting agent emtansine (TDM1) or the topoisomerase-I inhibitor deruxtecan (T-DXd). T-DM1 and T-DXd are approved for patients with HER2-positive metastatic breast cancer after disease progression on a trastuzumab-based regimen [3,31,32]. Necela et al. studied the association between FOLR1 and HER2 expression by analyzing the distribution of *FOLR1* mRNA expression using RNA-sequence data from multiple patient cohorts across three breast cancer subtypes: estrogen receptor-positive (ER+), HER2-positive (HER2+), and triple-negative (TNBC) tumors. Although their analysis revealed that high *FOLR1* expression was not limited to TNBC tumors, this type of tumor was significantly associated with increased expression of *FOLR1* mRNA compared with ER+ and HER2+ tumors [33]. The findings of that report agree with our results, as shown in Figure 1a, which show that various *FOLR1* expression levels across breast cancer cell types.

In our in vitro study, cell sensitivity to MORAb-202 was clearly associated with the expression of FOLR1 in breast cancer cell lines. However, in the orthotopic xenograft tumor mouse model, the growth of the MCF7 tumor was significantly suppressed despite a low FOLR1 expression level and resistance to MORAb-202 in vitro. Contrary to the in vitro condition, FOLR1 expression was observed in MCF7 tumor tissue by IHC analysis, suggesting that FOLR1 expression was induced by certain conditions in the in vivo model that was not provided in vitro. It has previously been reported that FOLR1 expression is upregulated under conditions of folate deficiency, as the increased metabolic requirements for folates to fuel nucleic acid synthesis and cellular growth induces a deficiency; subsequent repletion results in the downregulation of certain genes, along with transcriptional changes [34,35,36,37]. However, it was not clear whether the tissues surrounding the MCF7 orthotopic xenograft tumors were folate-deficient. Therefore, further studies are required to elucidate the mechanisms of FOLR1-upregulation in MCF7 tumors. Although MORAb-202 inhibited MCF7 orthotopic xenograft tumor growth, the remaining MCF7 tumor was larger than the T47D tumors 7 d after MORAb-202 administration, as observed by H&E staining of whole tumors (Figure 6e1 and Figure 7e1). These results suggest that high-FOLR1-expressing T47D orthotopic xenograft tumors shrank faster than MCF7 tumors and that the induction of FOLR1 might occur slowly in the latter.

We found that MORAb-202 affected cell cycle distribution, with G2-M arrest and subsequent apoptosis induction, exhibiting the well-known effects of eribulin [16,38]. Our results indicate that eribulin was released from MORAb-202 through linker degradation by lysosomal protease, and then the unleashed eribulin arrested the cell cycle at the G2-M phase in FOLR1-positive T47D cells. Moreover, MORAb-202 can be designed to eradicate not only FOLR1-positive cells but also to kill the surrounding cells through the bystander killing effect. Using a transwell co-culture system, we demonstrated that MORAb-202 decreases cell proliferation significantly in FOLR1-negative MCF7 cells that are co-cultured with HCC1954 cells in a medium supplemented with MORAb-202. Therefore, we suggest that MORAb-202 could be beneficially applied to treating tumors with heterogeneous target expression. T-DM1, one of the ADCs approved for patients with HER2-positive breast cancer, is conjugated to trastuzumab via a non-cleavable thioether linker, and the payload has a low level of cell permeability; therefore, the bystander effect is not observed in this case [39]. Conversely, the ADC T-DXd shows the bystander effect because of the high membrane permeability of the payload and owing to the conjugation of the payload with trastuzumab via a cleavable linker [39]. Therefore, the mechanism of MORAb-202 may be similar to that of T-DXd.

Cell sensitivity to MORAb-202 is clearly associated with FOLR1 protein and mRNA expression levels in breast cancer cell lines but not in NSCLC cell lines. The latter exhibit relative sensitivity to eribulin compared to MORAb-202 in vitro. It has been suggested that NSCLC cell lines possess driver mutations or gene amplification alterations that are not associated with FOLR1, such as the epidermal growth factor receptor (EGFR)-activating mutation found in the PC-9, HCC827, H1650, HCC4006, and H1975 cell lines; the RAS mutation in the PC-14 and A549 lines; and MET amplification in the NCI-H441 and ABC-1 lines [40]. These driver mutations or gene amplifications might confer MORAb-202 resistance to those cell lines. The receptor internalization and recycling mechanisms may differ between wild-type and mutated receptors [41,42]. Therefore, exposure to MORAb-202 might not be sufficient to kill such altered cells, owing to a low rate of either FOLR1 endocytosis-mediated internalization or linker degradation.

Recently, Monteiro et al. reported using two fluorescence-based techniques (flow cytometry and fluorescence microscopy) to study FOLR1 trafficking in living breast cancer cells from lines T47D, MDA-MB-231, and MCF7. They found that T47D internalized a greater quantity of FOLR1 than MDA-MB-231 or MCF7 and showed a higher recycling rate than MCF7 cells. Moreover, MCF7 cells displayed very low expression and functional levels of FOLR1 [43]. These findings agree with ours, showing that T47D cells are the most sensitive to MORAb-202, and MCF7 cells are the most resistant.

This study has several limitations. MORAb-202 is an ADC comprising an anti-FOLR1 antibody conjugated to a payload of eribulin via a cleavable linker. This linker should be enzymatically unleashed in the lysosome, releasing eribulin into intra- and intercellular spaces. However, this study lacks the direct determination of eribulin in intercellular spaces. Moreover, the mechanisms of FOLR1 upregulation in MCF7 orthotopic xenograft tumors requires further study. We determined that FOLR1 expression was associated with the inhibitory effect of MORAb-202 in breast cancer cell lines; however, as mentioned above, the rate of FOLR1 internalization or linker degradation should be considered by multidirectional studies.

## 5. Conclusions

We characterized the effect of MORAb-202, which is a novel ADC that comprises the anti-FOLR1 antibody conjugated to eribulin via a cleavable linker, on breast cancer and NSCLC cell lines. MORAb-202 sensitivity was clearly associated with protein and mRNA FOLR1 expression in breast cancer cell lines but not in NSCLC cell lines. MORAb-202 induced G2-M cell cycle arrest and subsequent apoptosis; these effects are identical to those of eribulin, indicating that eribulin was successfully released from MORAb-202 to intracellular spaces. In addition, the eribulin released into HCC1954 cells diffused through cell membranes into intercellular spaces and then killed the neighboring MCF7 cells in HCC1954-MCF7 co-culture systems in a bystander killing effect. Our in vivo experiment showed that the growth of T47D orthotopic xenograft tumors, which are high-FOLR1 expressing, is significantly suppressed under MORAb-202 treatment. Similarly, the growth of orthotopic xenograft MCF7 tumors was significantly suppressed, although MCF7 cells express low levels of FOLR1 in vitro. We attribute this result to the induction of FOLR1 in tumor tissue. The underlying mechanism remains unknown, and further studies are needed to elucidate it. Our results indicate that MORAb-202 is a potential ADC for FOLR1-positive tumors. Appropriate selection of cancer patients is required in future clinical trials of MORAb-202, along with the multilateral evaluation of FOLR1 expression, internalization, and degradation.

## Data Availability

Not applicable.

## Supplementary Materials

The following are available online at https://www.mdpi.com/2073-4468/10/1/6/s1, Figure S1: FOLR1 expression was quantified and determined using ImageJ software (NIH), Figure S2: the effect of MORAb-202 and eribulin in FOLR1-positive breast cancer cells.
