# Supplementary figures and images for: Novel Anti-FOLR1 Antibody–Drug Conjugate MORAb-202 in Breast Cancer and Non-Small Cell Lung Cancer Cells
Source: Antibodies (Basel). 2021 Feb 1;10(1):6. doi: 10.3390/antib10010006 (PMC7930947; doi:10.3390/antib10010006)

Supplementary Figure S1

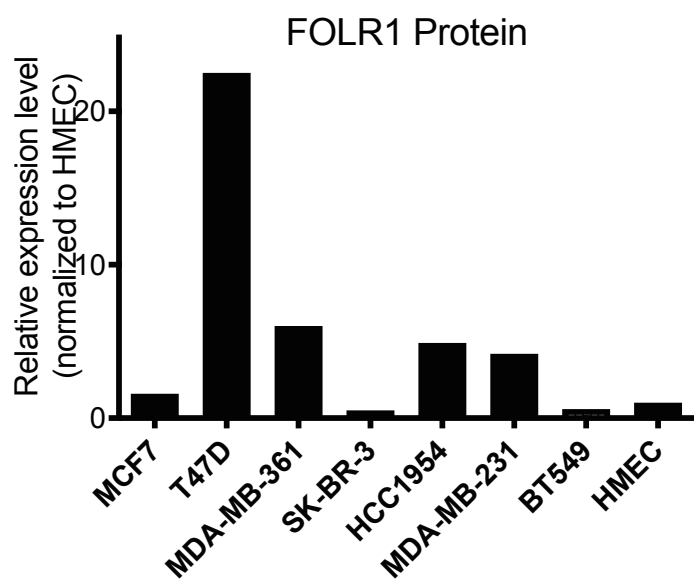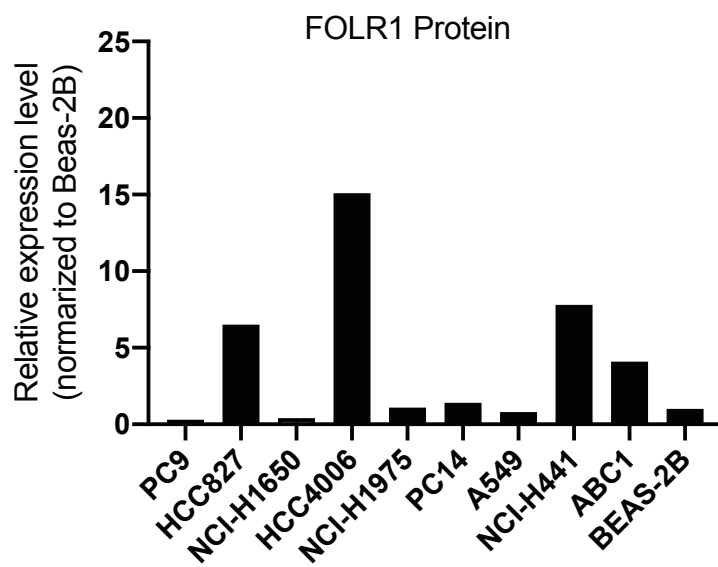

Supplement: Supplementary file 1 [file antibodies-10-00006-s001.zip › Supplementary figure 1.pdf]

Supplementary Figure S2

T47D

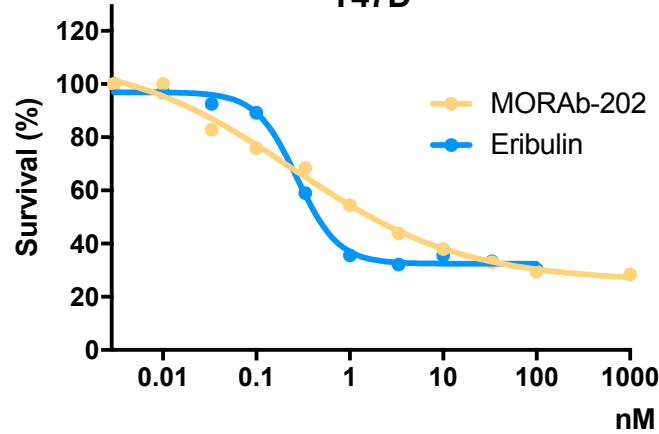

MDA-MB-361

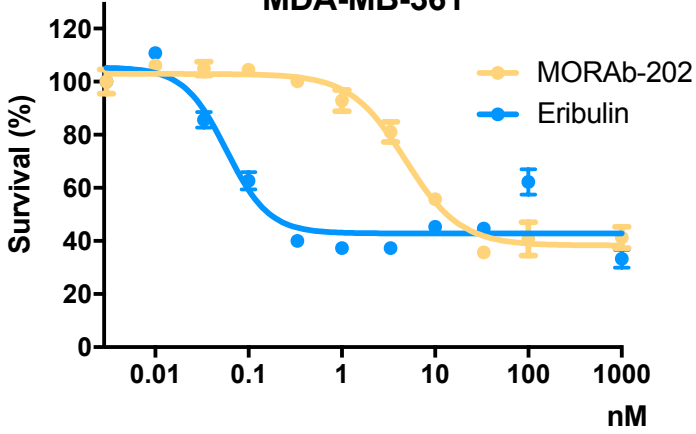

HCC1954

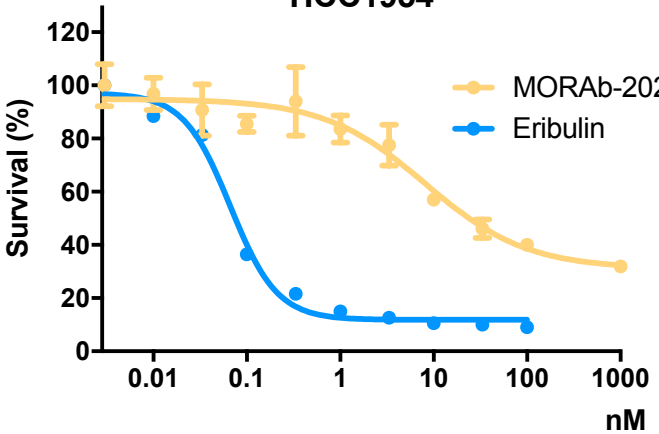

MDA-MB-231

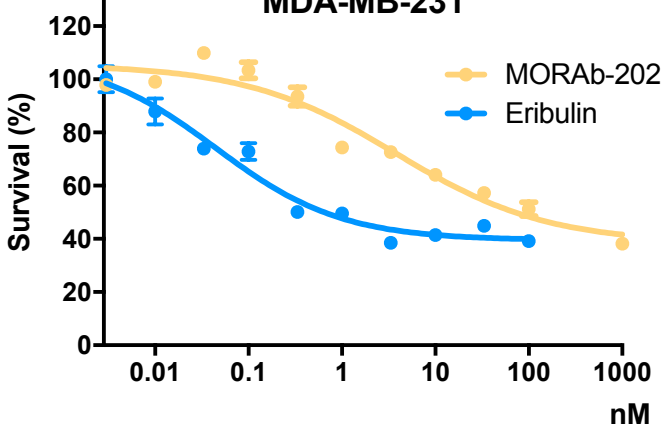

Supplement: Supplementary file 1 [file antibodies-10-00006-s001.zip › Supplementary figure 2.pdf]
